# Menopause, skin and common dermatoses. Part 2: skin disorders

**DOI:** 10.1111/ced.15308

**Published:** 2022-10-26

**Authors:** Erin Kamp, Mariha Ashraf, Esra Musbahi, Claudia DeGiovanni

**Affiliations:** ^1^ Department of Dermatology University Hospitals Sussex NHS Foundation Trust Brighton UK

## Abstract

In this second part of a four‐part review, we examine the effect of menopause on the skin. Menopause and the associated hypo‐oestrogenic state have implications for the structure and function of the skin. We performed a literature review to investigate the impact of the menopause on common dermatoses. There is evidence that oestrogen is implicated in transepidermal water loss and reduction in dermal collagen. There are associations with menopause and multiple common dermatoses, including xerosis and pruritus, hidradenitis suppurativa and psoriasis. Menopause has a clear impact on the skin and common dermatological conditions. Further research to understand the mechanisms and explore therapeutic options is needed.

## Abstract 2

Click here for the corresponding questions to this CME article.

## Introduction

Menopause, which starts on average between the ages of 45 and 55 years, is associated with falling oestrogen levels. The impact of the low oestrogen state of menopause has implications on the structure and function of the skin, with as many as 64% of women attending menopause clinics reporting skin problems (Table 1).^1^ Oestrogen receptors (ERs) are seen throughout the skin, with the highest density in the face, genital region and legs.^3^ ER‐β is predominantly seen on the skin, and reduction in circulating oestrogen is associated with a fall in ER expression.^4^ In this article we explore the impact of menopause on the skin and common dermatoses.

**Table 1:** Common dermatoses reported by menopausal women.^1^, ^2^

General dermatoses
Pruritus
Hyperhidrosis
Impaired wound healing
Xerosis
Eczema
Hair disorders
Hirsutism
Female pattern hair loss
Genital dermatoses
Lichen planus
Lichen sclerosis
Oral dermatoses
Lichen planus
Burning mouth syndrome
Xerostomia

## Search strategy

The Cochrane Library, National Institute for Health and Care Excellence Evidence database and the Turning Research into Practice database were searched from 2001 to 2021. In total, 116 original research articles were found on menopause in dermatology, 56 of which related to the skin and medical dermatoses. Individual searches were performed for specific queries related to our paper.

## Changes in skin structure after menopause

### Epidermis and sebaceous glands

The role of oestrogen within the epidermis is yet to be fully elucidated. However, the use of transepidermal oestrogen has been demonstrated to reduce transepidermal water loss (TEWL) and improve skin barrier function.^1^

The use of combined oestrogen and progesterone hormone replacement therapy (HRT) has been associated with an increase in sebum production and skin surface lipids.^5^ However, sole use of oestrogen is associated with reduction in the number and size of sebaceous glands and associated reduction in skin sebum levels.^6^

### Collagen

A rapid decline in skin collagen is seen following menopause, with nearly a third lost in the first 5 years and a decline of 2.1% per year over the 15 years following menopause.^1^ It has been clearly demonstrated that rather than being related to chronological age, collagen loss is related to postmenopausal age.^4^

Oestrogen HRT has been shown to increase dermal collagen levels to premenopausal states and can work prophylactically in preventing further collagen loss.^4^ An increase in collagen content and dermal thickness has been demonstrated as early as 3 months after starting treatment, and this is reproducible regardless of oestrogen administration route.^7^

### Elastin

Elastin degeneration increases with menopause and manifests clinically as slack skin and increased wrinkling.^4^ Studies on the efficacy of HRT are conflicting, and whereas the use of systemic oestrogen does not appear to affect elastin, some studies have demonstrated an increase in fibre thickness and total elastin with topical oestrogen when used on sites such as the buttocks and abdomen.^8^, ^9^, ^10^ Further research into the use of topical oestrogen HRT on facial skin is required.

## Common dermatoses after menopause

### Flushing

Flushing is seen in around 75% of perimenopausal and menopausal women and generally responds to oestrogen therapy.^8^ Reported treatment options are listed in Table 2. Symptoms occur secondary to vasodilation of the dermal and subcutaneous blood vessels, possibly due to a loss of peripheral vascular control associated with oestrogen deficiency.^8^, ^13^, ^14^

**Table 2:** Treatment options for menopausal flushing.^11^, ^12^

Hormonal
Oral oestrogen 0.3–1.25 mg daily
Other options include transdermal patch, vaginal rings and vaginal cream
Nonhormonal
Paroxetine 7.5 mg daily
Venlafaxine 36.5–75 mg daily
Desvenlafaxine 50–100 mg daily
Citalopram 10 mg daily
Escitalopram 10–20 mg daily
Gabapentin 300–900 mg three times daily
Pregabalin 10–50 mg three times daily
Behavioural therapy
Paced respiration training
Relaxation response training

### Xerosis, pruritus and dermatitis

A large observational study reported that eczematous eruptions, including allergic contact dermatitis and asteotic eczema, were the most commonly reported dermatoses in perimenopausal and menopausal women.^15^ One of the key pathophysiological players in dermatitis is a loss of barrier function with increased TEWL.^16^ As oestrogen therapy has been shown to reduce TEWL, it is possible that the hypo‐oestrogenic state could be implicated in increased TEWL and subsequent dermatitis.^6^

Pruritus is also frequently seen in postmenopausal women, commonly with associated xerosis. In addition to oestrogen HRT, general measures have been reported to be beneficial (Table 3).^1^

**Table 3:** General management options for postmenopausal pruritus.^1^

Low‐pH emollients at least once daily
Nails kept short
Use of humidifiers
Reduced bathing time
Avoidance of irritants (including soap)

### Acne

Acne, commonly seen in adolescence, is becoming increasingly recognized in adults. It typically presents with open and closed comedones, papules, pustules and inflammatory nodules. Rosacea is an important differential diagnosis; however, the presence of comedones in acne, unlike rosacea, is a helpful sign in differentiating between the two conditions.^17^ The pathophysiology of menopausal acne is unclear; however, an imbalance between oestrogen and androgens, leading to a relative hyperandrogenic state, is thought to be implicated.^17^ Therapeutic options include standard topical treatment, antiandrogenic therapy, including spironolactone, and isotretinoin.^17^

### Rosacea

Rosacea occurs frequently within the general population, and is particularly common in women.^18^ There is very little literature on the relationship between menopause and rosacea; however, menopausal flushing is known to exacerbate or even precipitate rosacea.^3^

### Hidradenitis suppurativa

There are clear associations with hidradenitis suppurativa (HS) and conditions associated with androgen excess, including acne vulgaris, hirsutism, and irregular menses.^19^, ^20^, ^21^ Flaring of HS with the menstrual cycle is reported to occur in as many as 63% of affected women.^22^ The impact of menopause on HS activity is unclear, with some studies reporting an improvement with menopause in 48% of women.^20^ This is in contrast with a more recent study of 279 women, which found that 39.5% of women reported their disease flaring and 44.2% reporting it as remaining stable with menopause.^23^

Hormone dysfunction is likely to play a role in the pathogenesis of HS; however, the exact mechanism remains unclear. A study found no significant difference in androgen receptor and ER activity in apocrine glands in patients with HS.^24^ Despite this, there does appear to be a benefit with antiandrogenic therapy.^22^, ^25^ There is anecdotal evidence that HRT can cause deterioration in HS symptoms.^22^ It is clear that further research is required to understand the role of hormones in HS and the therapeutic options available.

### Psoriasis

Psoriasis has been reported to improve, and occasionally clear, during pregnancy and to flare during the postmenopausal period.^26^ It is thought that reduced oestrogen leads to reduced inhibition of the T helper 1 cell‐mediated pathway, which is key in psoriasis pathogenesis.^27^ However, the findings of psoriasis flaring with menopause have not always been reproduced, with a large observational study in 2015 showing no significant changes in psoriasis with menopause.^28^

### Keratoderma climactericum

Keratoderma climactericum is a palmoplantar keratoderma associated with menopause and affecting the feet more severely.^29^ Reported treatments are listed in Table 4.

**Table 4:** Reported treatment options for keratoderma climactericum.^3^, ^29^, ^30^

Topical oestradiol 0.05%
25–40 urea creams
Etretinate 0.78 mg/kg/day^a^

^a^ Etretinate is no longer available; acitretin is a metabolite of etretinate.

### Pigmentation

There is a clear association with melasma and oestrogen, demonstrated by its association with pregnancy and oral contraception. There is no association with the menopause; however, there are cases of melasma developing following treatment with HRT.^4^

Up to 80% of women with extrafacial melasma are postmenopausal, but the pathogenesis remains unclear.^31^ Acquired bilateral melanosis (ABM) of the neck has been described in Asian perimenopausal women.^32^ The aetiology of ABM is unknown and response to treatment is poor.^32^

Although poikiloderma of Civatte is commonly recognized as occurring in women during the perimenopausal or menopausal period, the role of hormones in the pathogenesis is unclear and HRT is not recognized as a treatment option.^33^

### Hyperhidrosis

The reported degree to which postmenopausal women are affected by hyperhidrosis varies significantly, from 30% to 80%.^1^ The cause is unclear but responds to treatment with HRT. Other treatment options are listed in Table 5.

**Table 5:** Nonhormonal treatment options for hyperhidrosis.^1^, ^34^, ^35^, ^36^

Localized
Aluminium chloride 20% at night
Topical glycopyrronium 2.4% daily
Iontopheresis
Botulinum toxin
Systemic
Propantheline 15 mg 2–3 times/day
Glycopyrronium 1–2 mg 2–3 times/day
Oxybutanin 2.5–10 mg once daily
Surgical
Endoscopic thoracoscopic sympathectomy

## Cutaneous adverse effects of hormone replacement therapy

The systemic risks associated with HRT use include venous thromboembolism, and breast and endometrial cancer.^37^ However, the risks are generally considered to be low and outweighed by the beneficial impact. They are also less common with topical preparations.

Cutaneous adverse effects (AEs) of HRT include hirsutism, acne and androgenic alopecia, and local reactions are also reported.^3^, ^38^ Systemic lupus erythematosus flares less frequently and with less severity following menopause, thus there is a theoretical risk of precipitating flares with HRT.^3^

## Conclusion

The menopause state has clear implications for the skin, from collagen thinning and reduced elasticity to an impact on common dermatoses. The impact of these changes on affected women should not be underestimated. There is evidence that HRT can reduce or reverse some of these changes. Other treatment options depend on the specific complaint but are generally within the remit of dermatologists. It is clear that further research is required on the role of menopause on the skin and common dermatoses and, importantly for affected women, the role of HRT and the other therapeutic options available to them.

**Learning points**

- Dermal collagen is lost quickly after menopause, with a nearly a third lost in the first 5 years following menopause.
- Some studies have demonstrated an increase in elastin fibre thickness with topical oestrogen.
- Flaring of HS with the menstrual cycle is reported in as many as 63% of women; however, evidence is conflicting as to the role of menopause in disease activity.
- The pathophysiology of menopausal acne is unclear; however, an imbalance between oestrogen and androgens, leading to a relative hyperandrogenic state, is thought to be implicated.
- Cutaneous AEs of HRT include hirsutism, acne and androgenic alopecia.

## Conflict of interest

The authors declare that they have no conflict of interest.

## Funding

Funding for open access was provided by the University of Sussex as CDG is an honorary clinical lecturer for the university.

## Ethics statement

Ethics approval and informed consent not applicable as this was a literature review.

## CPD questions

### Learning objective

To gain knowledge on menopause, associated skin disorders and the impact of hormone replacement therapy.

### Question 1

Which of the following statements about the dermis and epidermis is correct?

(a) Dermal collagen loss is associated with chronological age rather than postmenopausal age.
(b) An increase in dermal collagen is seen only with oral oestrogen therapy.
(c) Use of topical oestrogen has been shown to reduce transepidermal water loss.
(d) Oral oestrogen has been demonstrated to increase elastin fibre thickness.
(e) Oestrogen therapy is associated with an increase in skin sebum levels.

### Question 2

Which of the following statements about menopausal flushing is correct?

(a) Flushing is reported infrequently in menopausal women.
(b) Paced respiration training is a recognized treatment option.
(c) Flushing usually responds well to progesterone replacement.
(d) Vasodilation of blood vessels in the underlying muscle is thought to be key in pathogenesis.
(e) Selective serotonin reuptake inhibitors (SSRIs) are not thought to be helpful in treatment of menopausal flushing.

### Question 3

Which of the following statements about hidradenitis suppurativa (HS) is correct?

(a) HS is reported to flare with the menstrual cycle in only a minority of women.
(b) Hormone dysfunction is not thought to be involved with the pathogenesis of HS.
(c) Hormone replacement therapy (HRT) has been reported to improve HS symptoms.
(d) HS is associated with acne vulgaris and irregular menses.
(e) Antiandrogenic therapy is generally not considered part of HS management.

### Question 4

Which of the following conditions has been reported not to flare with menopause?

(a) Acne.
(b) Hidradenitis suppurativa (HS).
(c) Hyperhidrosis.
(d) Melasma.
(e) Pruritus.

### Question 5

Which of the following statements about acne and rosacea is correct?

(a) Acne is a condition of adolescence and reported only rarely in adults.
(b) There is minimal overlap in the clinical presentation of acne and rosacea.
(c) Menopausal flushing is not known to exacerbate either condition.
(d) Although useful in managing acne in teenagers and young adults, isotretinoin is not used in menopausal acne.
(e) A relative hyperandrogenic state is thought to play a part in development of menopausal acne.

## Instructions for answering questions

This learning activity is freely available online at http://www.wileyhealthlearning.com/ced

Users are encouraged to

- Read the article in print or online, paying particular attention to the learning points and any author conflict of interest disclosures.
- Reflect on the article.
- Register or login online at http://www.wileyhealthlearning.com/ced and answer the CPD questions.
- Complete the required evaluation component of the activity.

Once the test is passed, you will receive a certificate and the learning activity can be added to your RCP CPD diary as a self‐certified entry.

This activity will be available for CPD credit for 2 years following its publication date. At that time, it will be reviewed and potentially updated and extended for an additional period.

## Data Availability

Not applicable.
